# Reshaping global health architecture: African health sovereignty as the foundation of global health equity and security

**DOI:** 10.4102/jphia.v17i1.1976

**Published:** 2026-04-24

**Authors:** Jean Kaseya, Nebiyu Dereje, Djoudalbaye Benjamin, Marta M. Terefe, Bethlehem Arega, Raji Tajudeen, Mosoka P. Fallah, Yap Boum, Claudes Kamenga, Shanelle Hall, Ngashi Ngongo

**Affiliations:** 1 Africa Centres for Disease Control and Prevention, Addis Ababa, Ethiopia

The overlapping epidemics that have unfolded across Africa in recent years – coronavirus disease (COVID-19), mpox, Marburg virus disease (MVD), Ebola and recurrent cholera outbreaks – have revealed structural weaknesses and inequities at the centre of the global health architecture. These emergencies exposed persistent inequities: wealthy nations securing vaccines and essential lifesaving medical countermeasures first, technology transfer blocked by intellectual property barriers, African genomic and epidemiological data used globally without meaningful benefit-sharing and regional institutions sidelined in decisions that determine access to lifesaving countermeasures.^1,2,3,4,5^ The African continent has been a consistent epicentre of high-impact epidemics, yet its institutions remain underrepresented in shaping how the world prevents and responds to them. This gap between epidemiological reality and political authority is no longer tenable. Africa’s lived experiences offer not only a diagnosis of what went wrong but also reveal why current mechanisms are not fit for purpose and outline the principles for a fair, functional and sovereignty-centred global health architecture.

The mpox outbreaks of 2022 and 2024–2025 made this clear yet again. While African countries struggled to access vaccines, diagnostics and therapeutics, supplies flowed rapidly to high-income countries as soon as mpox appeared in their populations – mirroring the early phases of COVID-19.^5,6^ The disparity occurred despite Africa having endemic mpox for decades and contributing much of the knowledge base that informed global countermeasure development, including vaccines that became inaccessible for African populations. These inequities persist not because the world lacks the tools to allocate countermeasures fairly, but because the current global system lacks binding rules that make fairness obligatory rather than aspirational. Mpox underscored that global health mechanisms built on goodwill will consistently reproduce structural imbalance. With those rules in place, the 2024–2025 outbreak would have been avoided if the populations in Eastern Democratic Republic of Congo (DRC) had access to vaccines, which could have limited the emergence of a new clade Ib that led to the expansion of mpox in more than 30 countries.^7^

The MVD outbreak in Rwanda in 2025 highlighted another dimension of inequity. Rapid containment was possible only because the government could immediately mobilise financing, surge clinical capacity and engage regional bodies such as the Africa Centres for Disease Control and Prevention (Africa CDC) promptly through the established incident management support team (IMST).^8,9,10,11^ The IMST provided cross-border coordination, genomic sequencing support and guidance, allowing Rwanda and neighbouring countries to synchronise surveillance and communication.^11^ But it also underscored how rare it is for African governments to have rapid access to emergency funds. Without immediate financing, the speed and scale of the response would have been considerably diminished. The Rwanda experience demonstrated the difference that timely, predictable and politically supported financing makes – and how slowly released global mechanisms undermine epidemic control.^9^

Cholera outbreaks across the Great Lakes region in the central and eastern African region, Southern Africa and the Horn of Africa illustrate the ways in which climate change, fragile water, sanitation and hygiene (WASH) infrastructure and global vaccine scarcity now intersect.^12^ El Niño-driven floods repeatedly contaminated water sources and displaced populations, triggering explosive outbreaks across multiple borders. Yet while countries sounded the alarm, the global oral cholera vaccine stockpile was insufficient to meet demand, forcing governments to use single-dose strategies and micro-targeting despite evidence that two doses provide more durable protection.^13,14,15^ Heads of State eventually called for a more robust political response to cholera, recognising that technical solutions cannot compensate for chronic underinvestment in climate-resilient water and sanitation systems. Without political leadership, cholera will remain a predictable, preventable catastrophe.^16,17^

These epidemics converged with a series of high-level dialogues convened by the African Union (AU), Africa CDC and Member States – including the landmark Addis Ababa Leaders’ Communiqué on Multilateral Cooperation for a Reformed Global Health Ecosystem.^18^ Collectively, these events produced an unprecedented continental consensus: health sovereignty is the foundation of health equity and security. Africa’s leaders called for a decisive transition away from an architecture characterised by dependency, fragmentation, unpredictable financing and externally driven priorities. They demanded reforms to ensure equity in access to countermeasures, strengthen regional manufacturing, elevate African leadership in global institutions and embed long-term resilience in primary health care and workforce systems.^18^ A core conclusion emerged: current global health mechanisms are not fit for purpose in a world defined by poly-emergency, decrease in official development assistance, climate instability and increasingly frequent pathogen spillover. Only a global architecture that centres equity, sovereignty and shared responsibility can meet the challenges ahead. To achieve this, several actions are urgently required.

**FIGURE 1:**
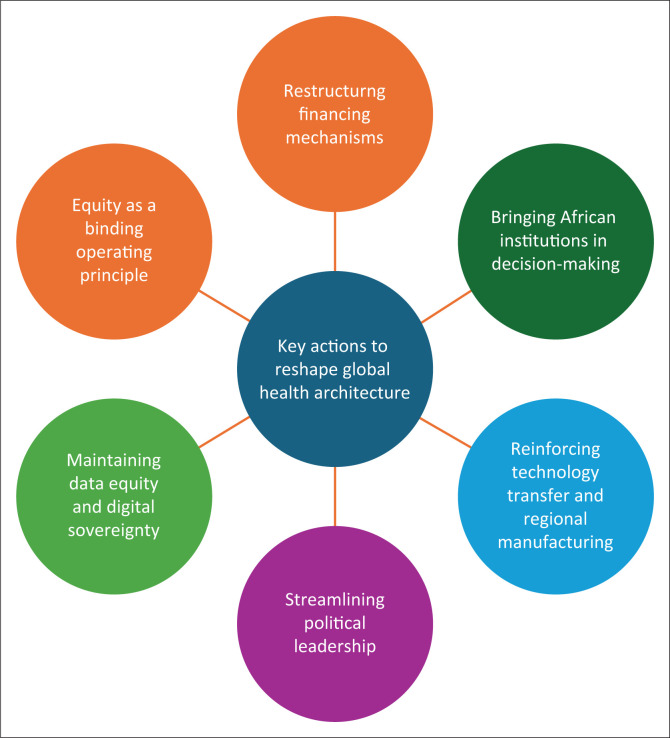
Key actions to reshape global health architecture.

We propose six interlocking reforms to realign global health architecture with epidemiological reality and African sovereignty (Figure 1).

Firstly, global health institutions must adopt equity as a binding operating principle. Allocation rules for vaccines, diagnostics, therapeutics and personal protective equipment must be pre-negotiated, transparent and automatically triggered when the World Health Organization (WHO) declares a Public Health Emergency of International Concern (PHEIC) or the Africa CDC declares a Public Health Emergency of Continental Security (PHECS). The ad hoc, politically mediated negotiations seen during COVID-19 and mpox, cannot continue to define access during emergencies. Likewise, advance market commitments must explicitly reserve purchasing quotas for African manufacturers, creating predictable demand that sustains manufacturing capacity rather than reinforcing dependence on a small group of suppliers.^19^

Secondly, global health financing mechanisms must also be restructured. Epidemic response should not depend on the speed of donor meetings. Global and regional surge financing mechanisms should release funds immediately upon the issuance of emergency declarations. The African epidemic fund could play a pivotal role in facilitating this.^20^ Moreover, countries should place innovative financing models that enable the release of surge finances during emergencies.^21,22^ Furthermore, global procurement systems must integrate regional manufacturing platforms into supply planning. This requires regulatory harmonisation, prequalification pathways tailored to diverse platforms, technical support from the African Medicines Agency and procurement diversification mandates for major funders.

Thirdly, global health governance must accurately reflect epidemiological realities by prioritising African institutions in decision-making. Africa CDC, the African Union Commission and regional economic communities should have legally guaranteed representation and voting rights within global health institutions, including WHO, Coalition for Epidemic Preparedness Innovations (CEPI), Gavi, the Global Fund and the Pandemic Fund. Their roles should extend beyond mere consultation; they should shape agendas, lead technical committees and influence funding decisions. The Pandemic Agreement and International Health Regulations (IHR) amendments must establish regional bodies as coordinating authorities, with the resources and authority commensurate with their responsibilities. Regional institutions are best suited to oversee cross-border surveillance, manage pooled procurement and offer technical support during crises. Strengthening governance also requires investments in African diplomatic, technical and legal capacities. Negotiating global agreements demands specialised expertise that many Member States cannot yet sustain. Regional institutions and national governments need dedicated units for treaty law, financing negotiations, global policy analysis and coordination of unified African positions. Without such capacity, Africa will continue to be represented rather than representing itself.

Fourthly, technology transfer and regional manufacturing must become non-negotiable pillars of global preparedness. The pandemic has demonstrated that controlling epidemics relies on timely access to vaccines, diagnostics, therapeutics and Infection Prevention and Control (IPC) supplies – items that Africa cannot reliably obtain from global markets during emergencies. Technology transfer must be a mandatory requirement for all publicly funded research and development (R&D), including that funded by high-income countries, philanthropic foundations and multilateral organisations. End-to-end transfer should include tacit knowledge, process engineering, regulatory support, workforce development and guaranteed market access. Financing models must support African manufacturing hubs from laboratory benches to commercial scale, with long-term concessional financing and procurement guarantees. A global technology transfer clearinghouse could accelerate local manufacturing by connecting innovators with regional producers, mediating licensing and resolving disputes when voluntary agreements stall.

Fifthly, data equity, digital sovereignty and open access knowledge exchange must be central to global cooperation.^23^ Africa Pathogen Genomics Initiative demonstrated that when African institutions lead sequencing and analysis, data sharing accelerates and response strategies become more precise. Yet too often, African data are extracted, analysed elsewhere and published without equitable benefit-sharing. FAIR (Findability, Accessibility, Interoperability and Reuse) principles must be adapted to emergencies with explicit protections for sovereignty and mechanisms for co-authorship, attribution and analytic access. Investments are needed in interoperable surveillance platforms, regional genomic networks, standardised biobanking, secure data-hosting infrastructure governed by African institutions and open-access knowledge exchange hubs. Data shared by Africa must be met with real-time analytics, capacity-building and transparent recognition. Emergency data-sharing protocols must be pre-negotiated to prevent delays during crises. The Africa Health Knowledge Hub, hosted by Africa CDC and supported by its Member States, serves as a centralised, open-access platform for curated, context-relevant health knowledge across the continent. It also enables real-time dialogue, collaboration, and expert exchange through interactive discussions and forums, thereby strengthening Africa’s voice and influence in shaping the global health architecture.^23^

Sixthly, global reform must be grounded in the political leadership already visible across the continent. The consensus emerging from the AU Summit side events reflects an unprecedented alignment: commitments to national ownership, financing aligned with country priorities, strengthened regional coordination, expanded African manufacturing, long-term workforce development and integrated approaches to climate- and humanitarian-driven health threats. The creation of a high-level Presidential Panel on Global Health Architecture Reform and a Ministerial Group representing all AU regions provides the political scaffolding needed to drive this agenda. Africa CDC’s mandate to serve as the Secretariat and coordinate follow-up offers continuity and institutional accountability. Global partners must now align with these structures rather than creating parallel ones.

The path forward is clear. A global health architecture that continues to rely on the charity of wealthy nations will fail once more. A system that concentrates manufacturing and decision-making power in only a few regions will sustain scarcity and cause delays. A data ecosystem that exploits Africa without offering reciprocity will undermine trust and weaken global intelligence. The pandemic agreement and its article on pathogen access and benefits-sharing should be critically scrutinised to bridge this gap. An architecture based on equity, sovereignty, shared responsibility and regional leadership is both practical and essential.^24,25^

Africa is already building this future through its health sovereignty agenda, expanded manufacturing initiatives, strengthened continental coordination and knowledge exchange and assertive engagement in global reform processes. The question that remains is not whether Africa is ready to lead – it is whether the global system is ready to follow, as a partner rather than a patron.^26,27^ The next pandemic will not wait for institutional reform. The world must act now – guided by Africa’s lived experience, political resolve and vision for a fairer, stronger and more resilient global health order.
